# A sensitive and reduced-cost culture medium for recovery of *Clostridioides difficile* isolates with reduced fidaxomicin susceptibility from stool

**DOI:** 10.1128/jcm.00068-26

**Published:** 2026-06-12

**Authors:** Claire E. Kaple, Jennifer L. Cadnum, Sarah N. Redmond, Annette L. Jencson, Munok Hwang, Hosoon Choi, Chetan Jinadatha, Curtis J. Donskey

**Affiliations:** 1 Department of Molecular Biology and Microbiology, Case Western Reserve University School of Medicine 196211, Cleveland, Ohio, USA; 2 Research Service, Louis Stokes Cleveland VA Medical Center 465630, Cleveland, Ohio, USA; 3 Department of Medicine, Case Western Reserve University School of Medicine 220786, Cleveland, Ohio, USA; 4 Department of Research, Central Texas Veterans Health Care System 525981 , Temple, Texas, USA; 5 Department of Medicine, Central Texas Veterans Health Care System 525981 , Temple, Texas, USA; 6 Department of Medical Education, College of Medicine, Texas A&M University 12332, Bryan, Texas, USA; 7 Geriatric Research, Education and Clinical Center, Louis Stokes Cleveland VA Medical Center 20083, Cleveland, Ohio, USA; Universitat Munster, Münster, Germany

**Keywords:** *Clostridioides difficile*, fidaxomicin, susceptibility, surveillance, culture medium

## Abstract

Emergence of resistance to fidaxomicin is a threat to current treatment algorithms for *Clostridioides difficile* infection (CDI), but current surveillance methods are labor-intensive and expensive. We tested the hypothesis that *C. difficile* Brucella agar (CDBA) media supplemented with 1 µg/mL fidaxomicin (CDBA-F1) would provide a sensitive, selective, and cost-effective method to screen stool specimens for *C. difficile* isolates with reduced fidaxomicin susceptibility (minimum inhibitory concentration >2 µg/mL). Clinical stool specimens testing positive for *C. difficile* (*N* = 126) were plated on CDBA and CDBA-F1; *C. difficile* isolates recovered were tested for fidaxomicin susceptibility using agar dilution. The sensitivity of CDBA-F1 in comparison to CDBA was 100% for recovery of isolates with reduced fidaxomicin susceptibility (eight of eight detected). Breakthrough growth of stool microbiota was common, but most breakthrough organisms were easily distinguishable from *C. difficile* by colony morphology. Use of CDBA-F1 could provide a sensitive and selective media for detection of *C. difficile* isolates with reduced susceptibility while substantially reducing labor and supply costs.

**IMPORTANCE:**

*C. difficile* isolates with reduced fidaxomicin susceptibility may emerge during CDI therapy and, in some cases, result in treatment failure. We report that supplementation of *C. difficile* selective media with fidaxomicin results in an effective, efficient, and reduced cost method to screen stool for isolates with reduced fidaxomicin susceptibility. Use of this approach could dramatically increase capacity to conduct surveillance for reduced fidaxomicin susceptibility in *C. difficile*.

## INTRODUCTION

*Clostridioides difficile* is the most common pathogen causing healthcare-associated infections in the United States (1). Fidaxomicin is recommended as a preferred treatment for *C. difficile* infection (CDI) because multiple trials have demonstrated improved sustained clinical response versus oral vancomycin (2). Although reduced fidaxomicin susceptibility (minimum inhibitory concentration [MIC] >2 µg/mL) has been rare in recent American and European surveillance studies (3–6), there have been several reports of clinical *C. difficile* isolates with reduced fidaxomicin susceptibility (MICs 2 to >64 µg/mL), and mutations in the *rpoB* or *rpoC* genes of RNA polymerase have been detected in some of these isolates (7, 8). Moreover, Redmond et al. (9) recently reported three patients with clinical failure of fidaxomicin therapy associated with emergence of *C. difficile* isolates with reduced fidaxomicin susceptibility. It is unclear if reduced susceptibility to fidaxomicin is emerging as an important concern because surveillance is limited, and culture and susceptibility testing is not included in diagnostic algorithms (2, 9).

One factor limiting surveillance efforts is that current methods for detection of reduced fidaxomicin susceptibility are labor-intensive and expensive. Stool specimens testing positive for *C. difficile* in clinical microbiology laboratories are cultured on *C. difficile* selective agar, and isolates are confirmed to be *C. difficile*. For all isolates recovered, MICs for fidaxomicin are determined using the reference agar dilution method recommended by Clinical and Laboratory Standards Institute (CLSI) (10). There is an urgent need for more efficient and cost-effective methods to screen for reduced fidaxomicin susceptibility. Antibiotic-impregnated selective media have been a useful tool for detection of other antibiotic-resistant pathogens (11). Here, we tested the hypothesis that *C. difficile* agar media supplemented with fidaxomicin would provide a sensitive, selective, and cost-effective method to screen for *C. difficile* isolates with reduced fidaxomicin susceptibility directly from stool.

## MATERIALS AND METHODS

### Study design

The facility’s institutional review board approved the study protocol (no. 1693200). Between 1 June 2020 and 31 December 2024, we screened a convenience sample of 126 stool specimens from patients with a positive *C. difficile* nucleic acid amplification test (NAAT) (Xpert *C. difficile*/Epi, Cepheid, Sunnyvale, CA) for isolates with reduced fidaxomicin susceptibility using standard culture media not containing fidaxomicin and the same media containing 1 µg/mL of fidaxomicin. Stool specimens from four patients included in the study were previously shown using standard methods to contain isolates with reduced fidaxomicin susceptibility that harbored mutations in RNA polymerase associated with reduced susceptibility (9). Medical record review was conducted to obtain information on prior exposure to fidaxomicin. The sensitivity and selectivity of the fidaxomicin-containing media were determined. We estimated the time, costs, and plastic and carbon footprints for screening with the standard method versus with the fidaxomicin-containing culture media. Polymerase chain reaction (PCR) ribotyping was performed for all isolates, and whole-genome sequencing was performed to identify mutations associated with reduced fidaxomicin susceptibility.

### Culture media

The Supplemental material includes details on formulation and preparation of the culture media. The base medium for culture of *C. difficile* was *Clostridium difficile* Brucella agar (CDBA) (12, 13). CDBA contains cycloserine 500 mg/L and cefoxitin 16 mg/L to inhibit growth of indigenous stool microbiota. The modified CDBA medium containing 1 µg/mL fidaxomicin (Tokyo Chemicals International, Tokyo, Japan) was termed CDBA-F1. Fidaxomicin (Tokyo Chemicals International, Tokyo, Japan) dissolved in dimethyl sulfoxide was added to liquid agar in petri dishes and allowed to solidify. The medium was used within 1 week of preparation and pre-reduced in a Whitley Workstation MG1000 anaerobic chamber (gas concentrations: 80% nitrogen, 10% carbon dioxide, 10% hydrogen) (Microbiology International, Frederick, MD) before use.

### Evaluation of the sensitivity and selectivity of the fidaxomicin-containing media

*C. difficile*-positive stool specimens obtained from the clinical microbiology laboratory were frozen at −80°C prior to analysis. The specimens were transferred to the anaerobic chamber and streaked for isolation onto CDBA and CDBA-F1 plates. The plates were incubated at 37°C for 48 h. The isolates recovered on selective media were plated on nonselective blood agar plates (Hardy Diagnostics, Santa Maria, CA) and identified using matrix-assisted laser desorption/ionization time-of-flight (matrix-assisted laser desorption/ionization-time of flight [MALDI-TOF]) mass spectrometry (details regarding MALDI-TOF are provided in the Supplemental material).

*C. difficile* isolates recovered from CDBA and CDBA-F1 were tested for fidaxomicin susceptibility using the reference agar dilution method (10). If multiple colonies consistent with *C. difficile* were recovered, one isolate was selected for susceptibility testing. The sensitivity of the CDBA-F1 plates for recovery of *C. difficile* isolates with reduced fidaxomicin susceptibility was calculated in comparison to the CDBA plates.

The use of fidaxomicin-containing media could potentially result in recovery of *C. difficile* isolates with reduced susceptibility that are present as a sub-population of all *C. difficile* present in stool specimens. To assess whether isolates with reduced fidaxomicin susceptibility were present as a sub-population, we plated dilutions of stool specimens that yielded these isolates concurrently on CDBA and CDBA-F1. The number of colony-forming units (CFUs) of *C. difficile* recovered from each of media type was compared. A greater number of colonies recovered on CDBA would suggest that isolates with reduced susceptibility may be present as a sub-population in a mixed population with predominantly susceptible isolates.

For plates that had breakthrough of colonies that were not consistent with *C. difficile*, representative distinct colony types were identified using MALDI-TOF, and pictures were taken. To assess selectivity of CDBA-F1, we calculated the percentage of plates with breakthrough colonies initially considered consistent with *C. difficile* based on appearance that were subsequently identified as other species or as *C. difficile* isolates that were susceptible to fidaxomicin. We also assessed growth of *Clostridium sporogenes* (American Type Culture Collection [ATCC] 11437), *Clostridium perfringens* (ATCC 131124), *Clostridium innocuum* (isolate recovered from a stool specimen), and vancomycin-resistant *Enterococcus faecium* strain C68 (14) on CDBA and CDBA-F1. The identified breakthrough isolates and the additional organisms evaluated for growth on CDBA-F1 were tested for susceptibility to fidaxomicin using agar dilution MICs.

### Fidaxomicin susceptibility testing

MICs for fidaxomicin were determined using the reference agar dilution method in accordance with CLSI recommendations (10) and as described by Thorpe et al. (5). The test medium was Brucella agar (Becton Dickinson, Sparksville, MD) supplemented with 5 mg/L hemin and 1 mg/L vitamin K1. Serial twofold dilutions of fidaxomicin (Tokyo Chemicals International) were added to liquid agar in petri dishes and allowed to solidify. The test isolates were diluted to a 0.5 McFarland standard, and a 2 µL inoculum was deposited on the agar in a final concentration of 10^4^ CFU per spot using a Steers replicator. The plates were incubated anaerobically at 37°C with MICs read after 48 h. The quality control strain was *C. difficile* ATCC 700057; the fidaxomicin MIC of ATCC 700057 was <0.25 µg/mL. Reduced susceptibility (RS) was defined as MIC >2 µg/mL, and resistance (R) was defined as MIC >16 µg/mL (9, 15).

### Work time, cost, and plastic and carbon footprint comparisons

We calculated the cost of supplies and reagents and the personnel time needed to conduct screening for *C. difficile* isolates with reduced fidaxomicin susceptibility using the standard method with CDBA plates not containing fidaxomicin versus CDBA-F1. The total time and cost for each method were calculated per 1,000 samples, estimating that 5% of samples may have reduced susceptibility (9).

The plastic footprint was calculated as the total amount of plastic waste generated using CDBA versus CDBA-F1 for screening per 1,000 *C. difficile* positive stool specimens screened. The carbon footprint was calculated based on the type of plastic in the materials used with a conversion rate of 1 kg of polystyrene to 2.7 kg of CO_2_ and 1 kg of HDPE to 1.7 kg of CO_2_ (16, 17).

### PCR ribotyping and whole genome sequencing

PCR ribotyping was performed for all isolates (9). Whole-genome sequencing was performed for the isolates with reduced fidaxomicin susceptibility as reported previously (9); for one reduced susceptibility isolate, Sanger sequencing of the *rpoB* and *rpoC* genes was performed in place of whole-genome sequencing. The Supplemental material provides detailed methods. Sequencing was performed on the NextSeq 550 Sequencing System (Illumina). *De novo* assembly of reads was done using SPAdes genome assembler (v.3.9), and a multilocus sequence type (MLST) was assigned. The *rpoB* and *rpoC* genes were detected using BLAST v2.11.0 utilizing reference genes against the assembled sequences. Trimmed reads were mapped to a *C. difficile* reference genome after dynamic trimming, and a dendrogram of the genome was created using advanced cluster analysis (similarity matrix) on Bionumerics software (v.7.6, Applied Maths).

### Data analysis

The sensitivity of CDBA-F1 for recovery of isolates with reduced fidaxomicin was calculated as the number of positive cultures for isolates with reduced fidaxomicin susceptibility recovered on CDBA-F1 divided by the number positive for CDBA screening. Student’s *t*-test was used to compare the numbers of *C. difficile* colonies recovered from CDBA-F1 in comparison to CDBA.

## RESULTS

### Sensitivity of CDBA-F1 for detection of isolates with reduced fidaxomicin susceptibility

**Fig 1:**
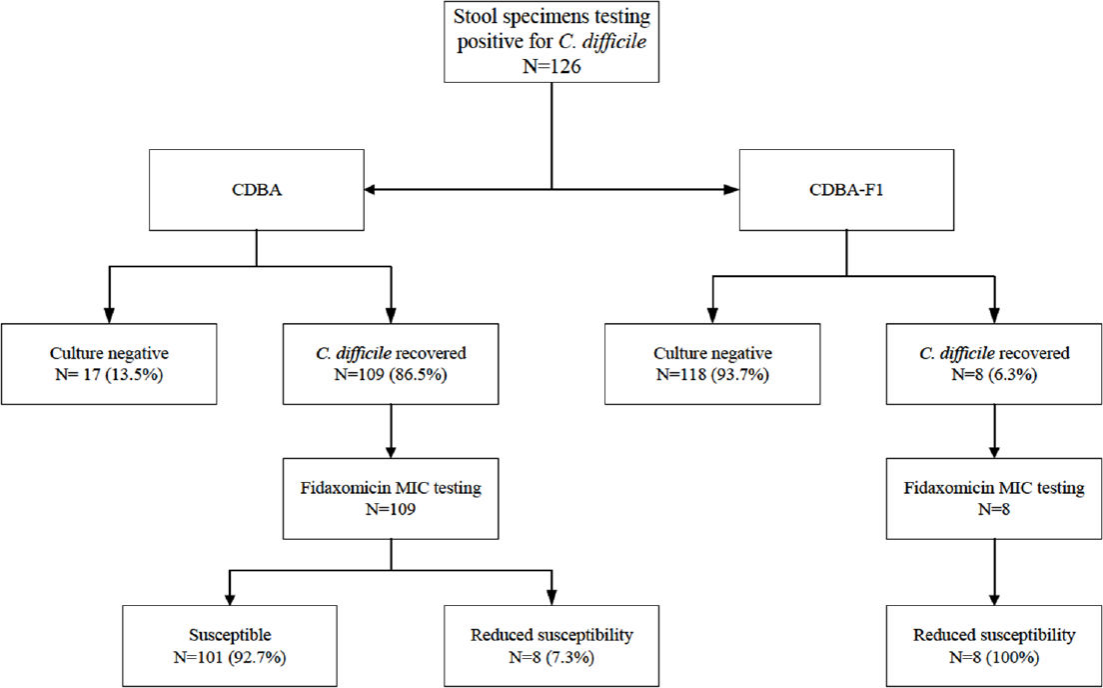
Flow diagram showing culture results for *Clostridioides difficile* using the two types of media. CDBA, *Clostridium difficile* Brucella agar; CDBA-F1, *C. difficile* Brucella agar containing 1 µg/mL fidaxomicin; MIC, minimum inhibitory concentration.

Figure 1 provides a flow diagram for the culture results for *C. difficile* using the two types of media. Of 126 stool specimens tested, 17 (13.5%) did not yield *C. difficile* growth on CDBA. Of the 109 *C. difficile* isolates recovered from CDBA, eight (7.3%) had reduced susceptibility to fidaxomicin, with MICs ranging from 16 to 128 µg/mL. For CDBA-F1, *C. difficile* colonies with reduced fidaxomicin susceptibility were recovered from the same eight stool specimens. All eight isolates recovered from CDBA-F1 had reduced fidaxomicin susceptibility by agar dilution, with MICs ranging from 16 to 128 µg/mL. Six of the eight (75%) patients with isolates with reduced susceptibility had received previous fidaxomicin treatment.

The sensitivity of CDBA-F1 for recovery of isolates with reduced fidaxomicin susceptibility was 100%. The number of colonies consistent with *C. difficile* recovered on CDBA-F1 did not differ from the number recovered on CDBA (mean, range; CDBA-F1 5.0 log_10_CFU/g stool, 2.5 to 6.0; CDBA 5.3 log_10_CFU/g stool, 2.3 to 6.6; *P* = 0.57).

### Selectivity of the culture media

**Fig 2:**
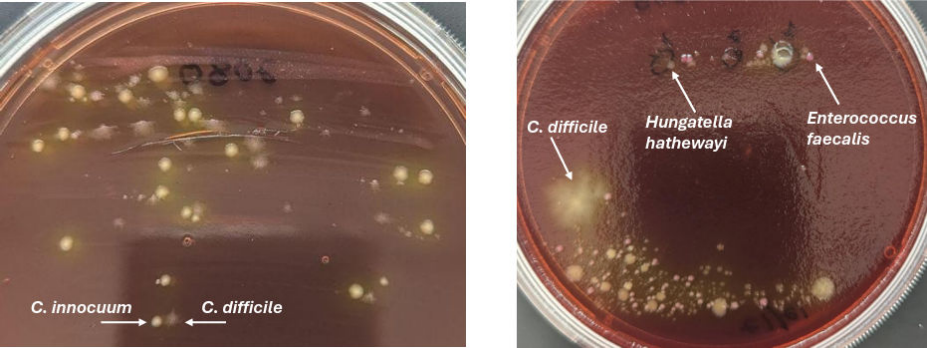
Pictures of typical colonies of *Clostridioides difficile* and of breakthrough organisms on *Clostridium difficile* Brucella agar (CDBA-F1).

No fidaxomicin-susceptible *C. difficile* isolates were recovered from CDBA-F1. On CDBA-F1 and CDBA, *C. difficile* colonies were yellow and diffuse or rough with filamentous edges or rarely smooth with rounded edges. Previous studies have demonstrated that some *C. difficile* strains display colony dimorphism with a mix of rough and smooth colonies (18). Approximately half of CDBA-F1 plates had breakthrough colonies that were not consistent with *C. difficile*. The most common breakthrough organisms were enterococci, which formed small, round, pink colonies that were easily distinguishable from *C. difficile*. All colonies consistent with *Clostridium* species were identified via MALDI-TOF. *C. innocuum* was recovered on approximately 10% of CDBA-F1 plates. *C. innocuum* colonies were typically smaller than *C. difficile* colonies and yellow to white in color with smooth, rounded edges (19). No colonies that were visually determined to be consistent with *C. innocuum* were identified as *C. difficile* by MALDI-TOF. Other species easily distinguishable from *C. difficile* that were recovered rarely (<5%) from CDBA-F1 plates included *Clostridium sporogenes*, *Klebsiella variicola*, *Staphylococcus epidermidis*, and *Hugatella hathewayi*. Figure 2 provides pictures of typical colonies of *C. difficile* and of breakthrough organisms on CDBA-F1.

**Fig 3:**
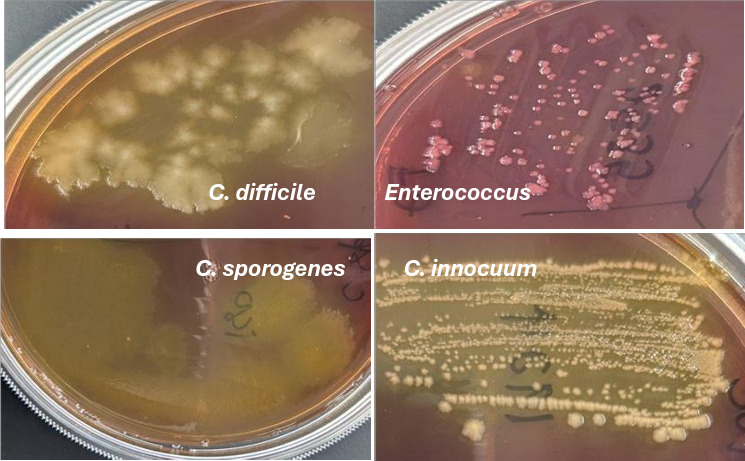
Pictures of *Clostridium sporogenes*, *Clostridium innocuum*, and *Enterococcus faecium* C68 control strains grown on *Clostridium difficile* Brucella agar (CDBA) containing 1 µg/mL (CDBA-F1).

The *C. sporogenes*, *C. innocuum*, and *Enterococcus faecium* C68 control strains that were used to assess selectivity grew on CDBA-F1, but *C. perfringens* did not. Figure 3 shows a 24-h culture of these organisms and *C. difficile* on CDBA-F1 (Fig. 3). The fidaxomicin MICs of the *C. innocuum*, *C. sporogenes*, and *E. faecium* C68 isolates were 16, 4, and 8 µg/mL, respectively.

### Work time and cost comparisons for use of CDBA versus CDBA-F2 for screening

Table 1 provides a comparison of the costs associated with use of CDBA agar and CDBA-F1 agar to screen 1,000 *C. difficile*-positive stool specimens for isolates with reduced fidaxomicin susceptibility. Use of CDBA-F1 reduced the calculated cost of supplies by $4,150 and the cost of labor by $4,925. The time required to process 1,000 stool specimens was reduced by 201 h.

**TABLE 1:** Comparison of the costs associated with use of two types of media to screen 1,000 stool specimens for *Clostridioides difficile* isolates with reduced fidaxomicin susceptibility^*a*^^,^^*b*^

	CDBA agar (no./amount, cost)	CDBA-F1 agar (no./amount, cost)
Supplies^*c*^		
CDBA or CDBA-F1 plates for initial screen (two samples/plate)	500 ($884)	500 ($949)
CDBA plate reisolate	450 ($796)	0 ($0)
Blood plates	500($555)	25 ($28)
Loops	3,900 ($1,997)	1,100 ($563)
Petri dishes for MICs	445 ($141)	24 ($8)
50 mL centrifuge tubes	445 ($531)	24 ($29)
Fidaxomicin	100 mg ($10)	20 mg ($0.5)
DMSO	100 mL ($70)	5 mL ($3.8)
Round bottom tubes	1,000 ($786)	50 ($39)
Total cost of supplies	$5,770	$1,620
Labor^*d*^	CDBA agar (hours/cost)	CDBA-F1 agar (hours/cost)
Make CDBA/CDBA-F1 plates	20	20
Plate on CDBA	17	17
Reisolate	5	0.8
Streak to blood plates	17	0.9
Make MIC plates	110	6
Set up MICs	55	3
Read MICs	16	0.8
Total time and cost of labor	249 ($6,125)	48 ($1,200)
Total cost supplies and labor	$11,895	$2,820

^*a*^ CDBA, *Clostridium difficile* Brucella agar; CDBA-F1, *Clostridium difficile* Brucella agar containing 1 µg/mL fidaxomicin; MIC, minimum inhibitory concentration; and DMSO, dimethyl sulfoxide.

^*b*^ Costs were calculated based on processing of 1,000 stool specimens, with 5% (50) having reduced fidaxomicin susceptibility defined MIC > 2 µg/mL.

^*c*^ Costs based on prices paid for purchase at the author’s institution.

^*d*^ Cost of labor calculated at $25 per hour.

### Comparison of estimated plastic and carbon footprints for use of CDBA versus CDBA-F2 for screening

For the screening method with CDBA agar and the method using CDBA-F1, the total plastic footprints were 22.7 (16.6 kg polystyrene and 6.1 kg HDPE) and 1.7 kg (1.7 kg polystyrene and 0 kg HDPE), respectively. For screening using the standard method with CDBA agar and the method using CDBA-F1, the calculated total carbon footprints were 54.6 and 4.5 kg of CO_2_, respectively. The savings of 50.1 kg of CO_2_ is equivalent to driving 251 mi in a vehicle with 30 mi per gallon of gasoline fuel efficiency.

### PCR ribotyping and sequencing analysis

The Supplemental material includes a table with the ribotyping results. There were 43 different ribotypes among the 101 fidaxomicin-susceptible *C. difficile* isolates, including 13 ribotypes with three or more isolates. The eight isolates with reduced fidaxomicin susceptibility included five different ribotypes, and all five were included among the 13 ribotypes with three or more fidaxomicin-susceptible isolates. Two ribotypes (014-020 and 002) were among the most common ribotypes reported in the United States in 2018 (20).

Table 2 shows the characteristics of the 8 *C. difficile* isolates with reduced fidaxomicin susceptibility. Seven of the eight isolates had *rpoB* or *rpoC* mutations previously reported to be associated with reduced fidaxomicin susceptibility, including a novel G266AA (R89K) *rpoC* mutation recently associated with clinical failure of an extended-pulsed fidaxomicin regimen (21). The isolate with no *rpoB* or *rpoC* mutations did not have mutations in the *marR* homolog CD22120, which has been associated with reduced fidaxomicin susceptibility (8). Two of the isolates (2399 and 2411) were recovered from a single patient with recurrent *C. difficile* who failed treatment with fidaxomicin, and three isolates (2396, 2419, and 2425) were recovered from a patient with recurrent CDI after treatment with fidaxomicin.

**TABLE 2:** Characteristics of the *Clostridioides difficile* isolates with reduced fidaxomicin susceptibility recovered from stool of patients with *C. difficile* infection^*a*^

Isolate number	Ribotype	Multilocus sequence type	*rpoB* mutation (amino acid substitution)	*rpoC* mutation (amino acid substitution)	Fidaxomicin minimum inhibitory concentration (µg/mL)
2161	014-020	224	T3428A (V1143D)	None	32
2175	002	8	None	None	16
1924	097	21	None	A265G (R89G)	16
2399	470	11	None	G266A (R89K)	32
2411	470	11	None	G266A (R89K)	32
2396	255	34	T3428A (V1143D)	None	128
2419^*b*^	255	ND	T3428A (V1143D)	None	128
2425	255	34	T3428A (V1143D)	None	128

^*a*^ Note. Two of the isolates (2399 and 2411) were recovered from a single patient with recurrent *C. difficile* infection who failed treatment with fidaxomicin, and three of the isolates (2396, 2419, and 2425) were recovered from another patient with recurrent *C. difficile* infection after treatment with fidaxomicin. ND, not done.

^*b*^ Sanger sequencing of *rpoB* and *rpoC* genes was used in place of whole-genome sequencing for this isolate.

## DISCUSSION

More efficient and cost-effective methods are needed to screen for *C. difficile* isolates with reduced fidaxomicin susceptibility. Our results demonstrate that CDBA supplemented with fidaxomicin could provide a sensitive and selective screening method for isolates with reduced fidaxomicin susceptibility. Although organisms other than *C. difficile* were often recovered on CDBA with or without fidaxomicin supplementation, most of these organisms were easily distinguished from *C. difficile*. Sequencing demonstrated that seven of the eight isolates with reduced susceptibility had *rpoB* or *rpoC* mutations that have been associated with reduced fidaxomicin susceptibility.

Our findings suggest that the use of CDBA-F1 could substantially reduce the amount of labor and supplies required to screen for isolates with reduced fidaxomicin susceptibility. For screening 1,000 *C. difficile*-positive stool specimens in our laboratory with a 5% reduced susceptibility rate, we estimated that use of CDBA-F1 rather than CDBA would reduce the number of work hours from 249 to 48 resulting in a $4,925 reduction in the cost of labor and a reduction of $4,150 in the cost of supplies. The overall reduction in the cost of supplies and labor would be $9,075 if CDBA-F1 were used rather than CDBA. The use of CDBA-F1 would also be associated with reduced carbon and plastic footprints.

Although screening with fidaxomicin supplemented media may provide a more efficient and reduced cost screening method, this method does have some limitations. First, the method requires the ability to perform anaerobic cultures and microbiological expertise. For many facilities, it may be more feasible to send stool specimens to a core laboratory for screening rather than setting up an in-house assay. Second, there is no established CLSI breakpoint for fidaxomicin susceptibility, and given that fidaxomicin achieves high stool concentrations, it is uncertain if there is a risk for treatment failure when isolates have relatively low MICs (e.g., 2–8 µg/mL mcg/mL). In our previous report, treatment failure occurred in three patients with infecting isolates having MICs of 16 or 32 µg/mL (9). Third, the culture method requires that the *C. difficile* organisms in NAAT-positive stool specimens be viable. In this study, 14% of stool samples that were frozen after testing positive by NAAT for toxin B genes were culture negative, possibly in part due to loss of viability during handling and/or freezing. Finally, as noted previously, breakthrough of non-*C*. *difficile* organisms occurred frequently and could potentially reduce sensitivity of the media if these organisms obscure *C. difficile* colonies. A heat or alcohol shock step could be added before plating stool specimens to reduce breakthrough by non-spore-forming organisms. *C. innocuum* with reduced fidaxomicin susceptibility was recovered from about 10% of CDBA-F1 plates and can be difficult to distinguish from *C. difficile* (21). MALDI-TOF or other confirmatory methods are essential to confirm that colonies are *C. difficile*.

Our study has some limitations. We only tested 126 total *C. difficile*-positive stool specimens from one healthcare facility, and the reduced susceptibility isolates only included five ribotypes. Additional studies are needed with larger numbers of stool specimens from multiple institutions. The reduced susceptibility isolates recovered had MICs ranging from 16 to 128 µg/mL. It is possible that the media may perform less well in settings with isolates with relatively lower MICs to fidaxomicin (i.e., MICs of 2 to 8 µg/mL). The estimated labor and cost savings were calculated based on an estimated 5% prevalence of isolates with reduced susceptibility in stool and labor time and costs for our facility. Other facilities may have lower or higher costs based on the expense of labor and supplies in their setting. The prevalence of reduced susceptibility is also likely to be relatively high in our facility based on high usage of fidaxomicin and routine testing of patients with fidaxomicin treatment failure (9). Finally, we did not test whether supplementation of CDBA with vancomycin, the other agent commonly used to treat CDI (2), would provide an effective means to screen stool specimens for isolates with reduced vancomycin susceptibility. We did not recover *C. difficile* isolates with reduced vancomycin susceptibility from vancomycin-supplemented media in a previous study in our facility (22).

## Data Availability

All genome sequences generated by this project have been submitted to NCBI (Bioproject ID PRJNA1199382). Reasonable requests for de-identified data will be honored by the corresponding author.

## SUPPLEMENTAL MATERIAL

Supplemental Material jcm.00068-26-s0001.docx Supplemental methods and results.

ASM does not own the copyrights to Supplemental Material that may be linked to, or accessed through, an article. The authors have granted ASM a non-exclusive, world-wide license to publish the Supplemental Material files. Please contact the corresponding author directly for reuse.
