# Supplementary material for: A sensitive and reduced-cost culture medium for recovery of Clostridioides difficile isolates with reduced fidaxomicin susceptibility from stool
Source: J Clin Microbiol. 2026 Jun 12;64(7):e00068-26. doi: 10.1128/jcm.00068-26 (PMC13343832; doi:10.1128/jcm.00068-26)
Supplement: Supplemental Material — Supplemental methods and results. [file jcm.00068-26-s0001.docx]

**Supplementary material**

**A sensitive and reduced-cost culture medium for recovery of *Clostridioides difficile* isolates with reduced fidaxomicin susceptibility from stool**

**1). Susceptibility testing methods**

MICs for fidaxomicin were determined using the reference agar dilution method in accordance with CLSI recommendations [1] and as described by Thorpe et al. [2]. The test medium was brucella agar (Becton Dickinson, Sparksville, MD) supplemented with 5 mg/L hemin and 1 mg/L vitamin K1. Fidaxomicin (Tokyo Chemicals International, Tokyo, Japan) was dissolved in dimethyl sulfoxide (DMSO). Serial 2-fold dilutions of fidaxomicin were added to liquid agar in petri dishes and allowed to solidify. The test isolates were diluted to a 0.5 McFarland standard and inocula were deposited on the agar in a final concentration of 10^4^ CFU per spot. The plates were incubated anaerobically at 37°C with MICs read after 48 hours. The quality control strain was *C. difficile* American Type Culture Collection (ATCC) 700057. Reduced susceptibility (RS) was defined as MIC >2 µg/mL and resistance (R) was defined as MIC >16 µg/mL [3].

**References**

1). CLSI. Methods for Antimicrobial Susceptibility Testing of Anaerobic Bacteria. 9th ed. CLSI standard M11. Wayne, PA: Clinical and Laboratory Standards Institute; 2018.

2). Thorpe CM, McDermott LA, Tran MK, et al. 2019. U.S.-based national surveillance for fidaxomicin susceptibility of *Clostridioides difficile*-associated diarrheal isolates from 2013 to 2016. Antimicrob Agents Chemother 63:e00391-19.

3). Baines SD, Wilcox MH. 2015. Antimicrobial resistance and reduced susceptibility in *Clostridium difficile*: Potential consequences for induction, treatment, and recurrence of *C. difficile* infection. Antibiotics (Basel) 4:267-98.

**2). Preparation of CDBA and CDBA-F1**

Media preparation was performed using standard methods [1,2]. Supplementary Table 1 shows the formulation of CDBA and CDBA-F1. The enriched brucella base was prepared by autoclaving for 15 minutes at 121°C followed by cooling to 50°C in a water bath. Sterile solutions of cycloserine (Sigma-Aldrich), cefoxitin (Sigma-Aldrich), sodium taurocholate, and lysozyme were prepared in distilled water and added to the cooled agar base after being filtered through a 0.2-µm-pore-size membrane (Corning, Corning, NY). The final concentration of sodium taurocholate was 0.1% (wt/vol). Agar was poured into petri dishes, cooled at room temperature, and allowed to solidify. Plates were pre-reduced in the anaerobic chamber for a minimum of 4 h before use.

**Supplementary Table 1. Formulation of *Clostridioides difficile* Brucella Agar (CDBA) and CDBA-F1**

CDBA ingredients per liter of water

Brucella broth powder ............................................................ 28.0 g

Vitamin K1 solution (1 mg/ml) .............................................. 1.0 ml

Hemin solution (5 mg/ml) ...................................................... 1.0 ml

Sodium bicarbonate solution (20 mg/ml).............................. 5.0 ml

Agar........................................................................................... 20.0 g

Mannitol.................................................................................... 6.0 g

Neutral red solution (1%)...................................................... 3.0 ml

Sodium taurocholate ............................................................... 0.5 ga

Lysozyme................................................................................... 5.0 mg

Cycloserine................................................................................500.0 mg

Cefoxitin.................................................................................... 16.0 mg

CDBA-F1 additional ingredient per liter of water

Fidaxomicin………………………………………………….1 mg/L

**Reference**

1). Nerandzic MM, Donskey CJ. 2009. Effective and reduced-cost modified selective medium for isolation of *Clostridium difficile*. J. Clin. Microbiol. 47:397-400. doi: 10.1128/JCM.01591-08.

**3). Preparation of fidaxomicin**

Fidaxomicin was dissolved in 100% dimethyl sulfoxide (DMSO) at a concentration of 10, 1, or 0.1 mg/mL. Precipitation of fidaxomicin occurred when the 10 mg fidaxomicin-100% DMSO solution was diluted in water, but no precipitation was noted when fidaxomicin dissolved in 100% DMSO was added directly to CDBA media. To prepare MIC plates and CDBA-F1, appropriate amounts of fidaxomicin stock solution dissolved in 100% DMSO were added to tubes containing 20 mL of warm liquid CDBA media at 65°C and mixed thoroughly. Supplementary Table 2 shows the stock fidaxomicin concentrations in 100% DMSO and the amount of stock solution added yield final concentrations. The tubes of liquid media were poured into petri dishes and swirled again to mix. The final concentrations (vol/vol) of DMSO in the MIC plates are shown in Supplementary Table 2. Preliminary testing demonstrated that these concentrations of DMSO did not inhibit growth of *C. difficile* (ATCC 700057).

**Supplementary Table 2. Stock fidaxomicin concentrations in 100% DMSO and the amount of stock solution added to 20 mL of CDBA media to yield final concentrations**

| Final concentration of fidaxomicin in plate (µg/mL) | Stock concentration of fidaxomicin in 100% DMSO (mg/mL) added to 20 mL of CDBA at 65°C | Volume of stock solution added (µL) | Final concentration of DMSO v/v % |
| --- | --- | --- | --- |
| 128 | 10 | 256 | 1.28 |
| 64 | 10 | 128 | 0.64 |
| 32 | 10 | 64 | 0.32 |
| 16 | 10 | 32 | 0.16 |
| 8 | 1 | 160 | 0.8 |
| 4 | 1 | 80 | 0.4 |
| 2 | 1 | 40 | 0.2 |
| 1 | 0.1 | 200 | 1 |
| 0.5 | 0.1 | 100 | 0.5 |
| 0.25 | 0.1 | 50 | 0.25 |
| 0.125 | 0.1 | 25 | 0.125 |

**References**

1). Wiegand, I., Hilpert, K. & Hancock, R. Agar and broth dilution methods to determine the minimal inhibitory concentration (MIC) of antimicrobial substances. Nat Protoc 2008;3:163–175. Available at: <https://doi.org/10.1038/nprot.2007.521>.

2). Clinical and Laboratory Standards Institute. 2018. Methods for antimicrobial susceptibility testing of anaerobic bacteria. 9th ed. CLSI standard M11. Wayne, PA: Clinical and Laboratory Standards Institute.

**4). MALDI-TOF and alternative methods to identify *C. difficile***

CDBA and CDBA-F1 were used to culture stool specimens known to be positive for *C. difficile* based on polymerase-chain reaction test positive for *C. difficile* toxin genes. Colonies consistent with *C. difficile* based on morphology were identified as *C. difficile* using the Bruker MALDI Biotyper. The Biotyper uses Matrix-Assisted Laser Desorption/Ionization Time-Of-Flight Mass Spectrometry (MALDI-TOF MS). The reference library includes over 4,320 species of microorganisms including *C. difficile*.

If MALDI-TOF MS is not available, alternative methods can also be used to confirm that colonies recovered on CDBA or CDBA-F1 are *C. difficile*. Colonies consistent with *C. difficile* are tentatively identified based on the characteristic odor and colony morphology. Gram stain can be used to confirm the presence of gram-positive rods. A positive proline-aminopeptidase test (PRO disc; Thermo Fisher Scientific) that detects L-proline aminopeptidase is useful to confirm C. difficile [1,2]. Commercial biochemical identification systems (e.g., API 20A, API ZYM) can also be used to confirm *C. difficile* [3].

**References**

1). García A, García T, Pérez JL. Proline-aminopeptidase test for rapid screening of *Clostridium difficile*. J Clin Microbiol. 1997 Nov;35(11):3007.

2). Park KS, Ki CS, Lee NY. Isolation and Identification of Clostridium difficile Using ChromID *C. difficile* Medium Combined With Gram Staining and PRO Disc Testing: A Proposal for a Simple Culture Process. Ann Lab Med. 2015 Jul;35(4):404-9.

3). ead CB, Ratnam S. Comparison of API ZYM system with API AN-Ident, API 20A, Minitek Anaerobe II, and RapID-ANA systems for identification of *Clostridium difficile*. Journal of Clinical Microbiology. 1988 Jan;26(1):144-146.

**5). Methods for whole genome sequencing and bioinformatic analysis**

Whole genome sequencing was performed as previously described [1]. After extracting genomic DNA using genomic DNA purification kit (NEB, Ipswich, MA), libraries were prepared using Nextera DNA prep Kit (Illumina, San Diego, CA). Sequencing was performed on the NextSeq 550 sequencing system (Illumina, San Diego, CA). *De novo* assembly of reads was done using St. Petersburg genome assembler (SPAdes genome assembler (v.3.9)) on the Bionumerics calculation engine [2] and a multilocus sequence type (MLST) was assigned based on the PubMLST database [3]. The *rpoB and rpoC* genes were detected using BLAST v2.11.0 utilizing reference genes against the assembled sequences.

Trimmed reads were aligned to a *C. difficile* reference genome using Burrows-Wheeler Aligner-MEM v0.7.17 and variances were filtered using stringent filtering criteria. Positions with at least one unreliable (N) base, ambiguous (non-ACGT) base, or gap were removed. Non-informative SNP positions (i.e, positions that contain SNPs in comparison with the reference sequence, but all sample sequences have the same base) were also removed. Each retained SNP had a minimum 5x coverage and was covered at least once in the forward and reverse direction. If an entry did not have sufficient coverage at a specific SNP position, the SNP was removed for that entry, resulting in an empty cell in the SNP matrix. The SNP position was removed from the SNP matrix if none of the entries had sufficient coverage for a given SNP. The minimum distance between SNPs was 12 bp.

The reference sequence was strain S-0253 (Genbank GCA_018885085.1) for clade 1 isolates and strain R20291 (Genbank NC_013316.1) for clade 2 isolates. A dendrogram of the entire genome was created using Unweighted Pair-Group Method with Arithmetic averages (UPGMA) cluster analysis on Bionumerics software (v.8.1, Applied Maths). Isolates were considered genomically related if they differed by <2 single nucleotide polymorphisms (SNPs). All isolates will be deposited in NCBI.

**References**

1). Gonzalez-Orta M, Saldana C, Ng-Wong Y, Cadnum J, Jencson A, Jinadatha C, Donskey C. 2019. Are many patients diagnosed with healthcare-associated *Clostridioides difficile* infection colonized with the infecting strain on admission? Clin Infect Dis 30;69:1801-1804.

2). Bankevich A, Nurk S, Antipov D, et al. 2012. SPAdes: A new genome assembly algorithm and its applications to single-cell sequencing. J Comput Biol 19(5):455–77.

3). Jolley KA, Bray JE, Maiden MCJ. 2018. Open-access bacterial population genomics: BIGSdb software, the PubMLST.org website and their applications. Wellcome Open Res;3:124. doi: 10.12688/wellcomeopenres.14826.1.

**6). Methods for Sanger sequencing of *rpoB* and *rpoC* genes**

Sanger sequencing of *rpoB* and *rpoC* genes was completed for 1 isolate (2419) that was inadvertently not sent for whole genome sequencing. The *rpoB* gene was amplified in a SYBR-based, two-step annealing qPCR assay using AGCTGGGTATCCTAGAGATGGT (forward) and CCCATATGATTTACAACATCTAGCTC (reverse) primers. The *rpoC* gene was amplified in a SYBR-based, single-step annealing qPCR assay using GGGAGTGCCACTGTGGTAAAT (forward) and ATCCAGTCTCTCCTGGATCAAC (reverse) primers. Purified PCR products were sent with *rpoB* GGTGAGTCTTTTGACAACAG (forward) and *rpoC* CGATCTTGGTGACATATCAAG (reverse) primers for Sanger sequencing.

**7). PCR ribotyping results**

**Supplementary Table 3. Distribution of polymerase chain reaction ribotypes for *Clostridioides difficile* isolates recovered from 109 stool specimens from patients with *C. difficile* infection (CDI), stratified by susceptibility to fidaxomicin***

| Ribotype | Fidaxomicin susceptible  (N=101) | Reduced fidaxomicin susceptibility (N=8) |
| --- | --- | --- |
| 106 | 16 (15.8) | 0 |
| 255 | 9 (8.9) | 3 (37.5) |
| 002 | 8 (7.9) | 1 (12.5) |
| 014-020 | 5 (5.0) | 1 (12.5) |
| 027 | 5 (5.0) | 0 |
| 012 | 5 (5.0) |  |
| 078-126 | 4 (4.0) | 0 |
| 485 | 4 (4.0) | 0 |
| 103 | 3 (3.0) | 0 |
| 015 | 3 (3.0) | 0 |
| 470 | 3 (3.0) | 2 (25.0) |
| 097 | 3 (3.0) | 1 (12.5) |
| 005 | 3 (3.0) | 0 |
| Other** | 30 (29.7) | 0 |

Data are shown as number (%)

*, reduced susceptibility was defined as MIC >2 µg/mL and resistance was defined as MIC >16 µg/mL (9,15)

**, ribotypes with <2 isolates
